# Plasmacytoid Dendritic Cell Response to CpG ODN Correlates with CXCL16 Expression and Is Inhibited by ox-LDL

**DOI:** 10.1155/2013/312590

**Published:** 2013-11-04

**Authors:** Mayda Gursel, Dennis M. Klinman, Ihsan Gursel

**Affiliations:** ^1^Department of Biological Sciences, Middle East Technical University, 06800 Ankara, Turkey; ^2^Cancer and Inflammation Program, National Cancer Institute, Frederick, MD 21702, USA; ^3^Department of Molecular Biology and Genetics, Bilkent University, 06800 Ankara, Turkey

## Abstract

Structurally distinct classes of synthetic CpG oligonucleotides (ODN) differentially activate human immune cells. K-type ODN trigger plasmacytoid dendritic cells (pDCs) to differentiate and produce TNF**α**. In contrast, D-type ODN stimulate large amounts of IFN**α** secretion from pDCs. The cell-surface receptor CXCL16 was previously shown to influence the nature and specificity of CpG ODN-induced immune activation. Here, we evaluated the expression and function of CXCL16 on pDC from healthy volunteers. We report that increased CXCL16 expression correlated with enhanced in vitro response exclusively to D-type CpG ODN. Conversely, enzymatic digestion of the receptor resulted in a decrease in IFN**α** production. Moreover, ox-LDL presence significantly inhibited D-ODN mediated IFN**α** production by pDCs. Coculture of enriched pDCs with the CXCR6 expressing Jurkat T cells decreased the activation threshold of these cells responding to D-ODN, suggesting that CXCL16/CXCR6 interaction may play an important role in modifying the response of pDCs to environmental danger signals.

## 1. Introduction

Synthetic oligodeoxynucleotides (ODN) containing unmethylated CpG motifs stimulate an innate immune response characterized by the production of cytokines, chemokines, and Ig by B cells, dendritic cells (DC), NK cells, and macrophages [1–4]. In humans, these motifs are recognized by B cells and plasmacytoid dendritic cells (pDC) that express Toll-like receptor 9 (TLR9) [5, 6]. Human PBMC recognize and respond to structurally distinct classes of CpG motifs [7–13]. Of these ODN classes, K type (also known as B class) phosphorothioate ODN express multiple TCGTT and/or TCGTA motifs that stimulate B cells to proliferate and secrete IL-6 and IgM and promote the survival, activation, and maturation of pDC in the absence of IFN*α* production [7, 8, 10]. In contrast, D type ODN (also known as A class) contain a phosphodiester purine/pyrimidine/CG/purine/pyrimidine motif capped at each end by a phosphorothioate polyG tail are poor stimulators of B cells [8–10]. However, D type ODN stimulate pDCs to produce high levels of IFN*α* [8–10]. Previous work has established that D but not K type ODN bind to the chemokine and scavenger receptor CXCL16 expressed on the surface of pDCs [14]. This interaction directs D type ODN into the recycling endosomal compartment, where TLR9-MyD88-IRF7 signaling pathway is activated, leading to robust IFN*α* production [15]. The 3rd class of CpG ODN, designated as C-type, contain one or two CpG motifs with a phosphodiester backbone at the 5′ end [11, 12]. C ODN also contains a palindromic sequence on a phosphorothioate backbone at the 3′ end and can induce proliferation of B cells and production of low amounts of IFN*α* from pDCs [11, 12].

CXCL16 functions as a scavenger receptor [16], a chemokine [17], and an adhesion [18] molecule, playing a prominent role in the pathogenesis of atherosclerosis [19] and psoriasis [20]. CXCL16 binds to the chemokine receptor CXCR6/Bonzo, expressed on the surface of CD4+ and CD8+ T cells [21]. Soluble to cell-surface expressed CXCL16 is controlled by the metalloproteinase ADAM10 that actively cleaves the membrane bound receptor [22]. This activity can be inhibited by the metalloproteinase inhibitor GM6001, thereby increasing the amount of the cell surface expressed CXCL16 [14, 22]. Conversely, treatment with o-sialoglycoprotease selectively digests the membrane-bound CXCL16 [14, 21]. In order to clarify the role of CXCL16 expression on human pDCs during CpG ODN-mediated immune activation, we modified the expression levels of this protein in human peripheral blood mononuclear cells prior to stimulation. Results indicate that preventing the cleavage of membrane-bound CXCL16 increased both the number of pDC expressing CXCL16 and their response to D-ODN. In contrast, digesting the membrane-bound CXCL16 reduced the number of pDC expressing CXCL16 and their response to D-ODN. Interestingly, our data indicated that circulating ox-LDL may have detrimental effect on pDC derived D-ODN mediated IFN*α* production, suggesting an adverse role during viral infection for individuals with elevated ox-LDL levels. Furthermore, we also show for the first time that coculture of purified pDCs with CXCR6 expressing Jurkat T cells decreased the threshold concentration of D-ODN mediated IFN*α* production. This effect was specific to CXCR6 as CCR5 expressing Jurkat cells proved to be ineffective. These results suggest an important role for CXCL16/CXCR6 interaction in modifying the response of pDCs to environmental danger signals.

## 2. Methods

### 2.1. Reagents

Endotoxin free ODN were purchased from IDT. Sequences of ODN used (5′ → 3′) were K3 CpG ODN, ATCGACTCTCGAGCGTTCTC; K3-flip control ODN, ATGCACTCTGCAGGCTTCTC; D35 CpG ODN, GGtgcatcgatgcaggggGG; D35-flip control ODN, GGtgcatgcatgcaggggGG; C type CpG ODN, TCGTCGTTTTCGGCGCGCGCCG. Bases shown in capital letters are phosphorothioate, and those in lower case are phosphodiester. All FITC-, phycoerythrin (PE)-, and PE-Cy5 conjugated antibodies except for BDCA-2 were purchased from Biolegend (London, UK). FITC- or PE-conjugated BDCA-2 was from Miltenyi Biotech (CA, USA). BDCA-4 magnetic bead based pDC isolation kit was from Miltenyi Biotec. Polyclonal goat anti-human CXCL16 (purified and biotin labeled) and its isotype matched control were from R&D Systems. 

### 2.2. Cells and Culture Conditions

PBMC (2–4 × 10^6^/mL) from healthy volunteers were obtained following informed consent and were cultured in RPMI 1640 containing 5% fetal calf serum (FCS), 50 U/mL penicillin, 50 *μ*g/mL streptomycin, 0.3 *μ*g/mL L-glutamine, 1 *μ*M nonessential amino acids, 1 *μ*M sodium pyruvate, 10 mM HEPES, and 10^-5 ^M 2-mercaptoethanol. Cells were stimulated for 24–48 h with 1–3 *μ*M ODN depending on the assay. In some experiments, magnetic bead enriched pDCs (100,000/well) were plated in 96-well flat-bottom plates. Cell-surface CXCL16 expression was modified/blocked by treating cells with 50 *μ*M GM6001 or 25 *μ*g/mL O-sialoglycoprotein endopeptidase (from Pasteurella haemolytica), 10 *μ*g/mL OxLDL, 10 *μ*g/mL LDL, recombinant IFN*γ* and TNF*α* (20 ng/mL each), or PMA/ionomycin (250 pg/mL/100 pg/mL) for 30 min at 37°C. Jurkat cells stably expressing CCR5 or CXCR6 were a kind gift from Dr. Keith Peden (FDA, CBER, Section of Retroviral Immunology) and were cocultured with enriched pDCs in 96-well U-bottom plates (1 : 1 ratio; 100,000 cells/well). 

### 2.3. Flow Cytometric Analysis

Cultured cells were washed in cold PBS, fixed, and stained as previously described [14]. Data was acquired (20,000–50,000 events) on a FACScalibur flow cytometer, and data were analyzed using the CELLQuest software (both from Beckton Dickenson, San Jose, CA). The following combination of antibodies were used in identification of pDCs: CD123(+)/BDCA-2(+).

### 2.4. ELISA

Ninety-six well microtiter plates (Millipore, Bedford, MA) were coated with antibodies that recognize human IFN*α* (PBL Biomedical Laboratories, New Brunswick, NJ), IP-10 (e-biosciences), or IL-6 (Biolegend) [14]. The plates were blocked with PBS-5% BSA. Supernatants from cultured cells were added, and their cytokine content quantitated by the addition of biotin-labeled anti-cytokine antibody followed by phosphatase-conjugated avidin and phosphatase-specific colorimetric substrate. Standard curves were generated using known amounts of recombinant human IFN*α*2a IP-10 or IL-6. All assays were performed in duplicate.

## 3. Results and Discussion

### 3.1. Metalloproteinase Inhibitor GM-6001 Enhances Cytokine Production Induced by D-ODN

**Figure 1:**
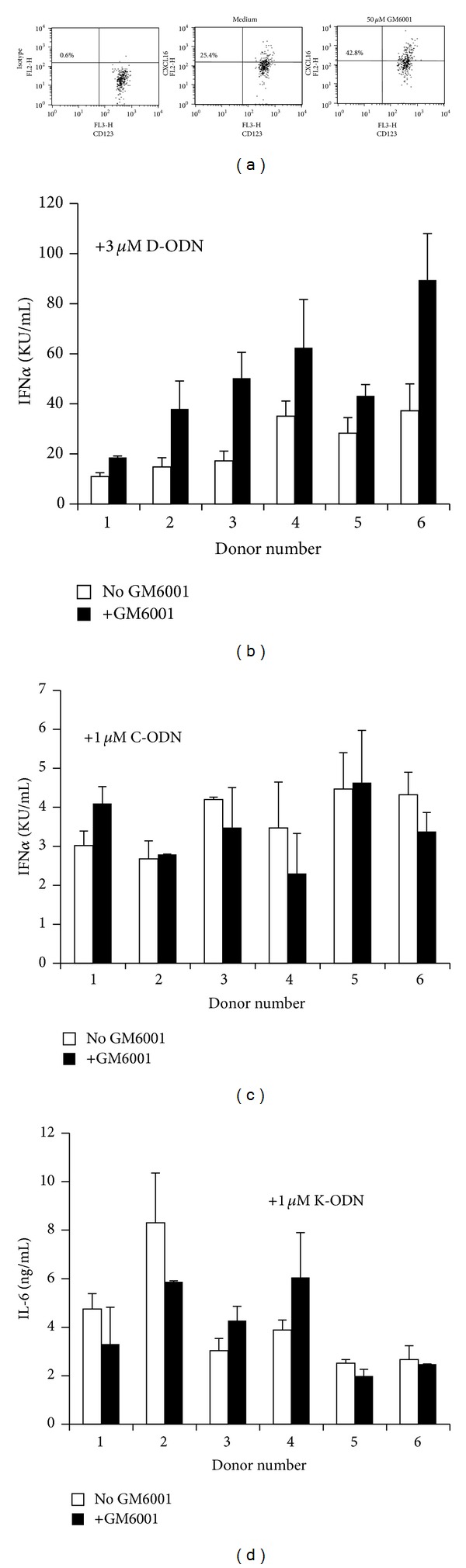
Metalloproteinase inhibitor GM-6001 enhances cytokine production induced by D-ODN.**  ** (a)**  **PBMC (4 × 10^6^/mL) were preincubated in the absence or presence of GM6001 (50 *μ*M) for 30 min, fixed, and stained for CXCL16 expression. Cells treated as in (a)**  **were washed and then stimulated with 3 *μ*M D-ODN (b), 1 *μ*M of C ODN (c), or 1 *μ*M of K-ODN (d) for 24 h. Cytokine production (IL-6 for K-ODN and IFN*α* for D and C ODN) was assessed from culture supernatants using ELISA. Response of 6 individual donors is shown.

Membrane-bound CXCL16 is expressed by professional antigen presenting cells (APC) such as pDCs and macrophages [14, 23]. Previous studies have demonstrated that the ratio of soluble to cell-surface expressed CXCL16 is controlled by the disintegrin-like metalloproteinase ADAM10 that actively cleaves the membrane bound receptor and that this activity can be inhibited by the metalloproteinase inhibitor GM6001, thereby increasing the amount of the cell surface expressed CXCL16 [22, 23]. To assess whether inhibition of ADAM10 would affect the response to the three classes of CpG ODN, PBMC from 6 donors were pretreated with GM6001 for 30 min and then stimulated with the D, C, or K type ODN. Exposure to the metalloproteinase inhibitor resulted in increased CXCL16 expression on pDCs (Figure 1(a)), whereas 24.6 ± 2.3 of untreated pDCs expressed cell-surface associated CXCL16 metalloproteinase inhibitor treatment caused a ~72% increase in surface expression levels for the protein (Table 1). GM6001 pretreatment also resulted in a significant increase in D-ODN responsiveness (~2-fold for individual donors, *P* < 0.05) (Figure 1(b) and Table 1) but had no effect on cells stimulated with C (Figure 1(c)) or K-ODN (Figure 1(d)). These results support the previous findings [14] and strengthen the case for CXCL16 involvement in D-ODN specific cellular activation. 

### 3.2. Cleavage of CXCL16 on the Surface of pDCs Reduces Cytokine Production Induced by D-ODN

**Figure 2:**
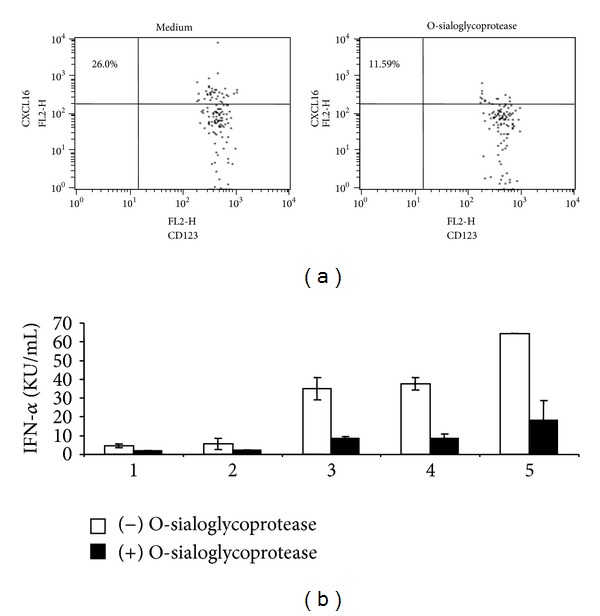
Digestion of CXCL16 on the surface of pDCs reduces cytokine production induced by D-ODN. (a) PBMC (4 × 10^6^/mL) were preincubated in the absence or presence of O-sialoglycoprotein endopeptidase (25 *μ*g/mL) for 30 min, fixed, and stained for CXCL16 expression. (b) Cells treated as in (a) were washed and then stimulated with 3 *μ*M D-ODN for 24 h. Cytokine production was assessed from culture supernatants using ELISA. Response of 6 individual donors is shown.

 Treatment of cells with the enzyme O-sialoglycoprotease was shown to cleave cell-surface expressed CXCL16 [22]. Treatment of PBMCs with this enzyme caused a significant reduction in pDC surface-associated CXCL16 protein levels (Table 1, *P* < 0.05 and Figure 2(a)). This decrease correlated with a significant reduction in D-ODN responsiveness (*P* < 0.05, Table 1 and Figure 2(b)). 

 These results indicate that factors that can modulate pDC associated cell-surface CXCL16 can affect the magnitude of the immune response to D-ODN. It remains to be seen whether CXCL16 also plays a role in modifying the immune response of pDCs during viral infections.

### 3.3. CXCL16/CXCR6 Interaction Reduces the Threshold of Activation in pDCs Responding to D-ODN

**Figure 3:**
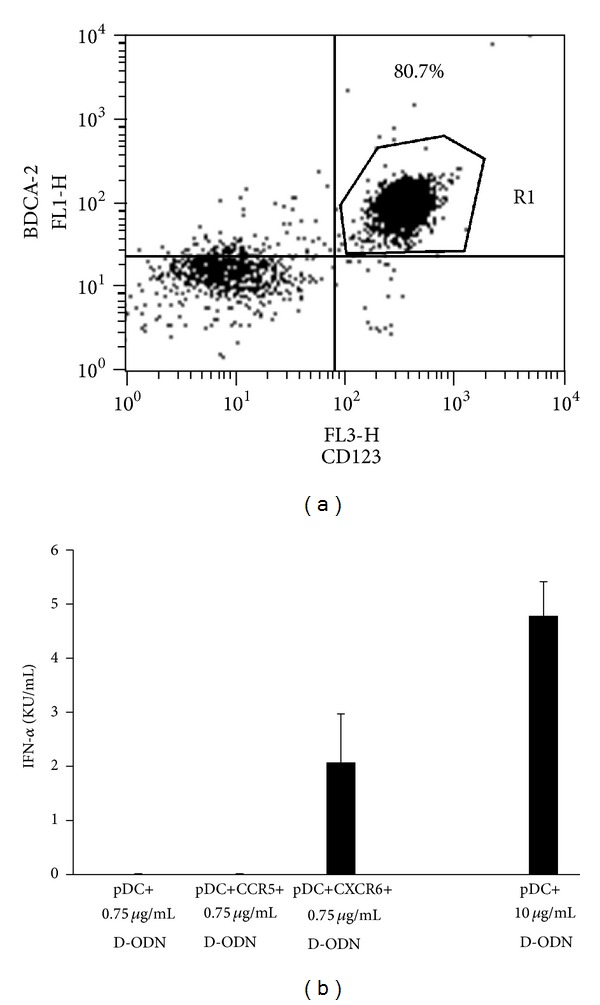
CXCL16/CXCR6 interaction reduces the threshold of activation in pDCs responding to D-ODN. (a) pDCs were enriched from human PBMCs using the BDCA-4 isolation kit as recommended by the manufacturer. Purity of cells was established by flow cytometry following staining with pDC associated cell-surface markers. (b) Enriched pDCs (100,000 cells/well) were incubated with CXCR6 or CCR5 expressing Jurkat T cells (1 : 1 ratio) in 96-well U-bottom plates. Cocultures were then stimulated with a suboptimal concentration of D-ODN (0.75 *μ*g/mL). pDC stimulated with optimal concentration of D-ODN (10 *μ*g/mL) served as a positive control. Cytokine production was assessed from culture supernatants 24 h later using ELISA. Average response of 3 different pDC preparations is shown (mean ± S.D).

 Transmembrane CXCL16 is composed of three domains: the chemokine domain that interacts with the receptor CXCR6, the glycosylated mucin-like stalk, and the cytoplasmic domain that contains a potential tyrosine phosphorylation and SH2-protein-binding site [21, 24]. Thus, the cytoplasmic domain may also play a role in cell signaling. To assess whether CXCL16 engagement on pDCs can modify the immune response to CpG ODN mediated immune activation, pDCs from 3 different donors were enriched using immunomagnetic separation (at least 80% pure as determined by pDC-specific marker staining, Figure 3(a)). Enriched pDCs were then incubated with CXCR6 expressing Jurkat T cells as a source to provide CXCL16 engagement. A separate set of pDCs were incubated with CXCR6 negative CCR5 positive Jurkat cells as negative control. The cocultures were then stimulated with suboptimal concentration of D-ODN that did not yield detectable levels of IFN*α* production (0.75 *μ*g/mL as determined in preliminary experiments). Results showed that coculture of purified pDCs with CXCR6 expressing Jurkat cells decreased the threshold concentration of D-ODN mediated IFN*α* production, enabling the cells to respond to an otherwise nonstimulating concentration of this ODN (Figure 3(b)). This effect was specific to CXCR6 since coculture with CCR5 expressing cells showed no such effect (Figure 3(b)). This result suggests that CXCL16/CXCR6 interaction may modify pDC responsiveness to environmental danger signals.

### 3.4. Oxidized LDL and Recombinant IFN*γ*/TNF*α* Modify Cytokine Production Induced by D-ODN

**Figure 4:**
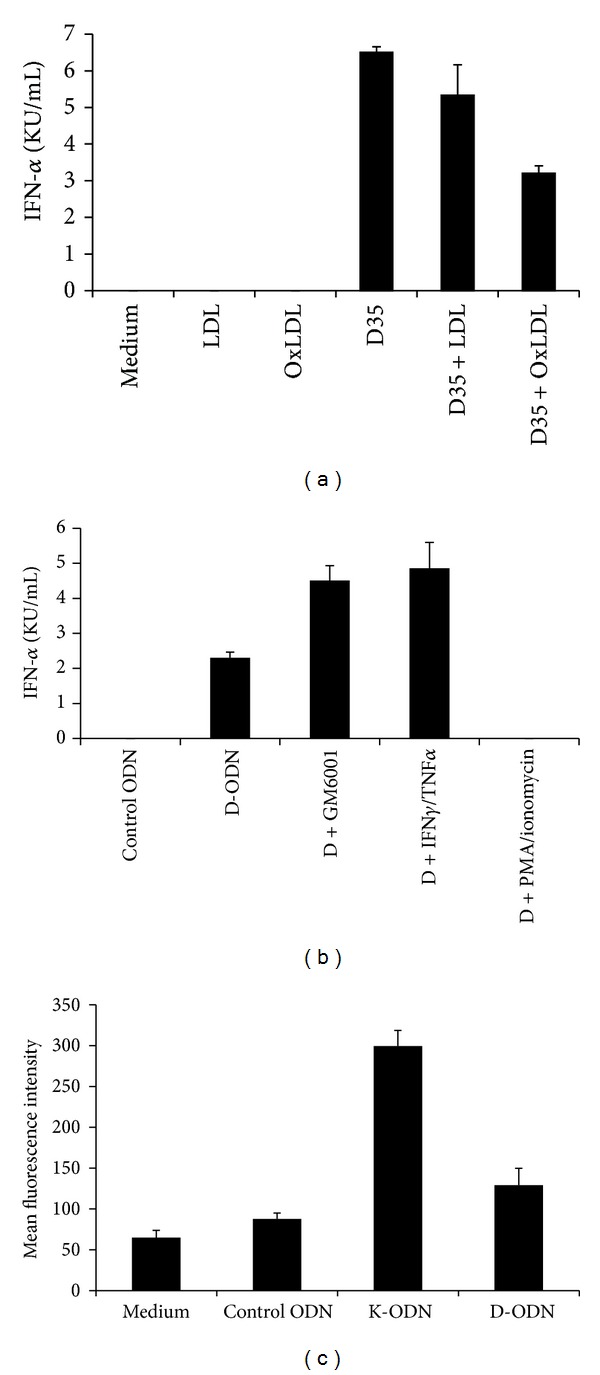
Oxidized LDL and recombinant IFN*γ*/TNF*α* modify cytokine production induced by D-ODN. (a)**  **Enriched pDCs (100,000 cells/well) were preincubated with LDL or OxLDL (10 *μ*g/mL each) and then stimulated with 3 *μ*M of D-ODN. Cytokine production was assessed from culture supernatants 24 h later using ELISA. Average response of 3 different pDC preparations is shown (mean ± S.D). (b)**  **Enriched pDCs were preincubated in the absence or presence of GM6001 (50 *μ*M), recIFN*γ*/TNF*α* (20 ng/mL each), or PMA/ionomycin (250 pg/mL/100 pg/mL) for 30 min at 37°C. Cytokine production was assessed from culture supernatants 24 h later using ELISA. Average response of 3 different pDC preparations is shown (mean ± S.D). (c)**  **PBMC (4 × 10^6^/mL) were preincubated in the absence or presence of 1 *μ*M Control ODN, 1 *μ*M K-ODN, or 3 *μ*M D-ODN for 48 h. Cells were then fixed and stained for CXCL16 expression. MFI of CXCL16 stained cells ± S.D of 3 different donors is shown.

We further examined the effect of scavenger receptor ligand oxidized low density lipoprotein (oxLDL) on D-ODN mediated immune activation. Preincubation of enriched pDCs with oxLDL resulted in ~50% reduction in IFN*α* production in samples stimulated with D-ODN, while native LDL showed very weak or undetectable inhibitory effect (Figure 4(a)). Hyperlipidemia and hypercholesterolemia are two prime risk factors in the development of atherosclerosis, and accumulating evidence suggests that DC functions may be hampered [25]. We demonstrated that increased levels of oxLDL may influence signaling pathways of pDC, thereby altering immune responses against pathogens. This result suggests that oxLDL levels may be of importance in modifying the pDC response during host resistance to microbial infections. 

Expression of CXCL16 is induced by the inflammatory cytokines IFN-gamma and TNF-alpha. These two cytokines synergize to upregulate both the soluble and the cell-surface associated CXCL16 [26]. To assess whether pDC response to D-ODN would be affected under conditions replicating an ongoing chronic inflammation, enriched pDCs were stimulated with the CpG ODN in the absence or presence of the recombinant cytokines IFN*γ*/TNF*α*. Results show that similar to GM6001, recombinant cytokine pre-treatment increased the D-ODN dependent IFN*α* production ~2-fold (Figure 4(b), *P* < 0.05). In contrast, PMA/ionomycin pretreatment which strongly downregulates CXCL16 expression [27] failed to induce cytokine production following D-ODN stimulation (Figure 4(c)).

K versus D-ODN differentially activate human cells to produce distinct cytokines (TNF*α* versus IFN*α*). To assess whether CXCL16 expression is altered during CpG ODN stimulation, PBMC were stimulated with K or D-ODN for 48 h, followed by staining for cell-surface expressed CXCL16. Cells stimulated with K-ODN expressed 3.4-fold more membrane-associated CXCL16 when compared to control ODN treated cells (Figure 4(c), *P* < 0.05). Conversely, D-ODN stimulated cells showed a modest increase (1.4-fold), suggesting that CXCL16 expression is not controlled by type I interferons.

In conclusion, we show that factors that can alter the extent of cell-surface CXCL16 expression can profoundly alter the immune response induced specifically by D-ODN and that CXCL16/CXCR6 interaction decreases the threshold concentration of D-ODN mediated IFN*α* production. This D-ODN sensitivity probably depends on the synergistic action of CXCL16/CXCR6 signaling cascade and more efficient delivery of D-ODN to endosome where TLR9 resides. 

## Figures and Tables

**Table 1:** Altering CXCL16 expression influences “D” ODN induced cytokine production.

	pDC expressing CXCL16		D-ODN induced IFN*α* production	
Treatment	% of all pDC	% change	KU/mL	% change
Untreated	24.6 ± 2.3		26.3 ± 17.8	
GM6001	43.1 ± 6.2*	↑72 ± 2*	50.3 ± 24.1*	↑115 ± 56*
O-sialoglycoprotease	9.6 ± 3.7*	↓55 ± 1*	6.6 ± 4.0*	↓70 ± 12*

PBMC from 5-6 different donors were treated with 50 *μ*M of GM6001 or 25 *μ*g/mL of o-sialoglycoprotein endopeptidase (from Pasteurella haemolytica) for 30 min at 37°C. The cells were then stimulated in vitro with 3 *μ*M “D” ODN for 24 h, and the production of IFN*α* determined by ELISA. The fraction of CD123/BDCA-2 double positive pDC expressing CXCL16 was determined before and after Rx. Treatment-induced changes were calculated for each donor independently and then averaged.

*P < 0.05.
